# Supplementary material for: Genome-Wide DNA Methylation Profiling as a Prognostic Marker in Pituitary Adenomas—A Pilot Study
Source: Cancers (Basel). 2024 Jun 13;16(12):2210. doi: 10.3390/cancers16122210 (PMC11201450; doi:10.3390/cancers16122210)
Supplement: Supplementary file 1 [file cancers-16-02210-s001.zip › CpG sites, DMPs and DMRs.pdf]

S3 – Gene overlaps by Clusters - DMPs and DMRs

Cluster	DMPs	Gene Set Description<br>(top ten)	Gene Overlap<br>(p-value)	DMRs	Genes
1	8873 (886 with mean diff over 0.3)	RHO GTPase cycle<br>RHOG GTPase cycle<br>RHOA GTPase cycle<br>RAC1 GTPase cycle<br>Phosphatidylinositol signaling system<br>Signaling by Rho GTPases, Miro GTPases and RHOBTB3<br>CDC42 GTPase cycle	13 (2.07e-7)<br>6 (1.27e-6)<br>7 (6.43e-6)<br>7 (2.53e-5)<br>5 (2.81e-5)<br>13 (3.38e-5<br>6 (8.8e-5)	6	CREB5<br>SDK1<br>PROSER2-AS1 PROSER2
2	325	Signaling by Receptor Tyrosine Kinases<br>G-protein mediated events<br>PDGFR-beta signaling pathway<br>Nitric Oxide Signaling Pathway<br>HS-GAG degradation<br>VEGF signaling pathway<br>Fc epsilon RI signaling pathway<br>Opioid Signalling<br>Ras signaling<br>Fc gamma R-mediated phagocytosis	9 (3.22e-6)<br>4 (7.24e-6)<br>5 (1.2e-5)<br>3 (1.46e-5)<br>3 (2.22e-5)<br>4 (2.83e-5)<br>4 (3.29e-5)<br>4 (5.49e-5)<br>5 (6.56e-5)<br>4 (7.07e-5)	2	ITM2C<br>SOCS2
3	3114 (1231 with mean diff over 0.3)	Pathways in cancer<br>PDGFR-beta signaling pathway<br>ErbB signaling pathway<br>ErbB signaling pathway<br>Osteoblast differentiation and related diseases	11 (2.58e-6)<br>7 (8.79e-6)<br>6 (9.96e-6)<br>6 (1.21e-5)<br>6 (5.62e-5)	107	GALNT17<br>GAS7<br>UBN1<br>USP7<br>NAT16<br>PLCXD2<br>NHSL3<br>GATA3<br>POLR1A<br>SLC7A7<br>ASIC2<br>POU2AF3<br>COLCA1<br>CLASRP<br>TRIM36

					IRX5<br>JAK1<br>ARPP21<br>AHR<br>CADM1<br>NCAM1<br>LDLRAD4<br>PREP<br>GLRA3<br>ETS1<br>RIPOR2<br>TTC7A<br>ATPAF1<br>EFCAB14<br>TNRC6B<br>NUDCD1<br>KCTD16<br>PDE3B<br>MEF2C<br>ECHDC3<br>FBLN1<br>NTM<br>PARD3<br>WDR25<br>PCNX2<br>TPRG1<br>ANO6<br>PTPRO<br>AXIN2<br>LGR4<br>LINC00681<br>LINC03019<br>RAC1<br>DIPK2A<br>KCNQ2

					SRGAP3<br>MLEC<br>AJAP1<br>SETBP1<br>IQGAP2<br>MSI2<br>GPM6A<br>TMEM108<br>KISS1R<br>SPATA31E1<br>NRP1<br>LINC02864<br>ROBO2<br>GLI2<br>CDC42<br>LINC01625<br>EYS<br>ZSWIM6<br>PUM1<br>LINC00473<br>SAMD11<br>ASB4<br>PTPRK<br>ATP13A3<br>SEMA6A<br>PEX5L<br>CACNA1G<br>AFG1L<br>CNGB3
4	674	Developmental Biology<br>Ectoderm differentiation<br>Pleural mesothelioma<br>Integration of energy metabolism<br>White fat cell differentiation<br>VEGFA VEGFR2 signaling	20 (3.72e-6)<br>7 (6.48e-6)<br>11 (1.1e-5)<br>6 (1.53e-5)<br>4 (1.73e-5)<br>10 (5.39e-5)	0	NA

		Circadian rhythm genes<br>ATM signaling in development and disease<br>1p36 copy number variation syndrome	7 (6.06e-5)<br>4 (6.83e-5)<br>5 (1.03e-4		
5	0	NA		2	LINC00886<br>MYO3A
