# Supplementary material for: Genome-Wide DNA Methylation Profiling as a Prognostic Marker in Pituitary Adenomas—A Pilot Study
Source: Cancers (Basel). 2024 Jun 13;16(12):2210. doi: 10.3390/cancers16122210 (PMC11201450; doi:10.3390/cancers16122210)
Supplement: Supplementary file 1 [file cancers-16-02210-s001.zip › Protocol for bisulfite conversion before DNA methylation analysis_cg.pdf]

Protocol for bisulfite conversion before DNA methylation analysis - based on Epitech® Plus DNA Bisulfite Kit

1. Bisulfite Mix inserted in Thermomixer for 5 minutes at 60 °C and 1400 RPM
2. Distribute Mastermix in MicroAmp 8 tube strip with attached domed caps.
3. Add 40 µl of DNA (see protocol above) to each well.
4. Insert bisulfite tray into Mini AMP thermocycler – run PCR program: BilsufiteConvers*

*PCR program: **BisulfiteConvers**		
Denat.	95°	5 min.
Incubate	60°	25 min.
Denat.	95°	5 min.
Incubate	60°	85 min. (1h 25 min.)
Denat.	95°	5 min.
Incubate	60°	175 min. (2h 55 min.)
Hold	20°	uendelig
Estimated time about 5 hours		

Purification of bisulfite-converted DNA:

1. Pipette 310 µl Buffer BL-carrier RNA mix into 1.5 ml Eppendorf Safe-Lock microtube.
2. Add Bilsufite converted DNA 140 µl.
3. Vortex and centrifuge briefly.
4. Add 250 µl ethanol 96% Ph Eur, BP.
5. Vortex and centrifuge shortly.
6. Transfer 700 µl of the content by pipette to a MinElute DNA spin columns placed in a 2 ml collection tube.
7. Centrifuge for 1 minute.
8. Discard the material in the collection tube and add 500 µl Buffer BW.
9. Centrifuge for 1 minute.
10. Discard the material in the collection tube and add 500 µl Buffer BD.
11. Incubate at room temperature for 15 minutes.
12. Centrifuge for 1 minute.
13. Discard the material in the collection tube and add 500 µl Buffer BW – Repeat this step twice.

14. Discard the material in the collection tube and add 250 µl ethanol 96 % Ph Eur, BP.
15. Centrifuge for 1 minute.
16. Discard the material in the collection tube and transfer MinElute DNA spin columns to new collection tubes.
17. Centrifuge for 1 minute.
18. Transfer MinElute DNA spin columns into 1.5 ml Eppendorf Safe-Lock microtube.
19. Add 15 µl Buffer EB to Eppendorf Safe-Lock microtube.
20. Incubate at room temperature for 1 minute.
21. Centrifuge for 1 minute.
