# Supplementary material for: Genome-Wide DNA Methylation Profiling as a Prognostic Marker in Pituitary Adenomas—A Pilot Study
Source: Cancers (Basel). 2024 Jun 13;16(12):2210. doi: 10.3390/cancers16122210 (PMC11201450; doi:10.3390/cancers16122210)
Supplement: Supplementary file 1 [file cancers-16-02210-s001.zip › Protocol for DNA methylation analysis - Illumina EPIC® with BeadChip and illumina iScan_cg.pdf]

Protocol for DNA methylation analysis - Illumina EPIC® with BeadChip and Illumina iScan

1. Before use of a new EZ DNA Methylation® Kit, add 24 ml 100 % ethanol and 6 ml M-Wash Buffer concentrate.
2. Turn on Eppendorf Thermomixer Comfort to 37 °C.
3. CT Conversion Reagent (750 µl Sigma Water and 210 µl M-Dilution Buffer added to a CT Conversion Reagent tube).
4. In a 1,5 ml Eppendorf tube, add M-Dilution Buffer and Sigma Water, and then centrifuge.
5. Incubate for 15 minutes at 37 °C.
6. Add 100 µl CT Conversion Reagent to each sample.
7. Transfer each sample (150 µl per sample) to a 96-well plate and close the lid.
8. Place the plate on a 2720 Thermal Cycler and run the PCR-program iscan bisulfit (volume input set to 100 µl).
9. The program lasts for approximately 16 hours.
10. For each sample, a Zymo-Spin® IC Column is placed in a collection tube.
11. Add 400 µl M-Binding Buffer to each column.
12. Centrifuge at 16100 xg for 30 seconds, then discard the fluid in the collection tubes.
13. Add 100 µl M-Wash Buffer to each column.
14. Centrifuge at 16100 xg for 30 seconds, then discard the fluid in the collection tubes.
15. Add 200 µl M-Desulphonation Buffer to each column and incubate for 15-20 minutes at room temperature.
16. Centrifuge at 16100 xg for 30 seconds, then discard the fluid in the collection tubes.
17. Add 200 µl M-Wash Buffer to each column.
18. Centrifuge at 16100 xg for 30 seconds, then discard the fluid in the collection tubes.
19. Place the columns in 1.5 ml Eppendorf tubes and add 12 µl M-Elution Buffer on the filter of each column.
20. Centrifuge at 16100 xg for 30 seconds for the DNA to wash out.
21. Restoration by ZR-96 DNA Clean & Concentrator®-5 Kit, add 192 ml 100 % ethanol to 48 ml DNA Wash Buffer.
22. Turn on the Hybex® Microsample Incubator and set to 37 °C.
23. Remove PPR (Primer Pre Restore) and AMR (AMP Mix Restore) from the freezer and thaw at room temperature. Vortex and centrifuge.
24. Transfer 8 µl of bisulfide-converted DNA to each well of a MIDI plate.
25. Add 4 µl 0.1 NaOH to each well and incubate for 10 minutes at room temperature.
26. Add 34 µl PPR to each sample.
27. Add 38 µl AMR to each sample, then close the plate with a rubber lid.
28. Turn the plate upside down 10 times and centrifuge at 280 xg for one minute.
29. Incubate MIDI plate in the Hybex® Microsample Incubator for one hour at 37 °C.
30. Thaw ERB (Elution Restore Buffer) and CMM (Convert Master Mix) at room temperature. Vortex and centrifuge.
31. Remove the MIDI plate from the incubator and centrifuge at 280 xg for one minute.
32. Remove and discard the rubber lid.
33. Place the Zymo-Spin® I-96 Plate from the ZR-96 DNA Clean & Concentrator®-5 Kit on top of the collection plate.
34. Add 560 µl DNA Binding Buffer to each sample on the MIDI plate.

35. Mix by pipette 5 times (up and down) each sample (approx. 650 µl) to the Zymo-Spin® I-96 Plate on top of the collection plate.
36. Centrifuge the Zymo-Spin® I-96 Plate on top of the collection plate at 2235 xg for two minutes, then discard the fluid in the collection tubes.
37. Add 600 µl DNA Wash Buffer (with added ethanol) to each well.
38. Centrifuge the Zymo-Spin® I-96 Plate on top of the collection plate at 2235 xg for two minutes, then discard the fluid in the collection tubes.
39. Place the Zymo-Spin® I-96 Plate on a new MIDI plate and add 13 µl ERB to each well.
40. Incubate for five minutes at room temperature.
41. Centrifuge the Zymo-Spin® I-96 Plate at 2235 xg for one minute for the DNA to wash out.
42. Seal the plate with Barseal® and incubate at 95°C for 2 minutes.
43. Immediately after incubation, place the plate in at bucket of ice for 5 minutes.
44. While still on ice, remove the Barseal® and add 10 µl CMM to each well. Close the plate with a rubber lid.
45. Vortex the plate on illumina® High-Speed Microplate Shaker at 1600 rpm, for 1 minute.
46. Centrifuge at 280 xg.
47. On the Hybex® Microsample Incubator set to 37 °C, incubate the plate for one hour.
48. Centrifuge at 280 xg.
49. Place the Zymo-Spin® I-96 Plate on top of the collection plate.
50. Remove and discard the rubber lid and add 140 µl DNA Binding Buffer to each well.
51. Mix by pipette 5 times (up and down) each sample (approx. 160 µl) to the Zymo-Spin® I-96 Plate on top of the collection plate.
52. Centrifuge the Zymo-Spin® I-96 Plate on top of the collection plate at 2235 xg for two minutes, then discard the fluid in the collection tubes.
53. Add 600 µl DNA Wash Buffer (with added ethanol) to each well.
54. Centrifuge the Zymo-Spin® I-96 Plate on top of the collection plate at 2235 xg for two minutes, then discard the fluid in the collection tubes.
55. Take a new MIDI Plate and mark it "MSA5".
56. Place the Zymo-Spin® I-96 Plate on top of the "MSA5" plate and add 10 µl Sigma Water on the filter to each well.
57. Incubate for five minutes at room temperature.
58. Centrifuge the Zymo-Spin® I-96 Plate on top of the "MSA5" plate at 2235 xg for one minute for the DNA to wash out.
59. Add 20 µl MA1 to each well.
60. Add 4 µl 0.1 NaOH to each well.
61. Close with a rubber lid.
62. Vortex the"MSA5" plate on illumina® High-Speed Microplate Shaker at 1600 rpm, for 1 minute.
63. Centrifuge at 280 xg.
64. Incubate for 10 minutes at room temperature.
65. Remove the rubber lid gently and add 68 µl RPM and 75 µl MSM to each well.
66. Close with the rubber lid.
67. Vortex the"MSA5" plate on illumina® High-Speed Microplate Shaker at 1600 rpm, for 1 minute.
68. Centrifuge at 280 xg.

69. Incubate the “MSA5” plate in the Illumina® Hybridization Oven for 20-24 hours at 37 °C.
70. Ensure the Hybex® Microsample Incubator is set to 37 °C.
71. Insert “MSA5” plate in incubator.
72. Defrost FMS to room temperature. Mix by manual turn at least 10 times
73. After incubation of “MSA5” plate for 20-24 hours, centrifuge the plate at 280 xg
74. Remove the rubber-lid from the MSA5 plate and add 50 µl FMS to each well.
75. Close MSA5 plate with the rubber-lid.
76. Vortex the plate on illumina® High-Speed Microplate Shaker at 1600 rpm, for 1 minute.
77. Centrifuge at 280 xg
78. Incubate MSA5 plate in the Hybex® Microsample Incubator for one hour at 37 °C.
79. Remove the rubber-lid and add 100 µl PM1 (at room temperature) in each well of MSA5 plate.
80. Close the rubber-lid and vortex the plate on illumina® High-Speed Microplate Shaker at 1600 rpm, for 1 minute.
81. Incubate the plate for five minutes on a termoblock at 37 °C.
82. Centrifuge at 280 xg and cool the centrifuge to 4 °C.
83. In a fume hood remove the rubber-lid and add 300 µl 100 % 2-propanol to each well.
84. Close the MSA5 plate with a new rubber lid.
85. Mix the content manually by turning the plate upside down a least 10 times.
86. Incubate for 30 minutes at 4 °C.
87. Centrifuge the plate at 2250 xg for 20 minutes at 4 °C.
88. Remove the plate from the centrifuge and discard the rubber lid.
89. Drain the plate for liquid upside down on a Tena Cellduk cloth. Tap the plate multiple times until all liquid is drained.
90. Still on the cloth upside down on a tube rack, rest the plate for 1 hour at room temperature. Wait until dry blue pellets appear in the bottom of the wells.
91. Turn on the Illumina® Hybridization Oven to 48 °C.
92. Preheat the Variant Temperature Sealer for 20 minutes.
93. Dissolve the RA1 reagent by manually turning it multiple times.
94. In a fume hood, add 30 µl RA1 to each well of the MSA5 plate containing DNA pellets.
95. Cover the MSA5 plate with a Seal Heat Foil with the blank side op and insert in the Variant Temperature Sealer. Release the handle and hold for 3 seconds.
96. Remove the plate and run a piece of paper over the Seal Heat Foil, so each of the 96 wells are marked on the foil. If not, repeat step 26.
97. Incubate the sealed MSA5 plate in the Illumina® Hybridization Oven for one hour at 48 °C.
98. Vortex the plate on illumina® High-Speed Microplate Shaker at 1800 rpm, for 1 minute.
99. Centrifuge at 280 xg.
100. Denature the MSA5 plate in the Hybex® Microsample Incubator at 95 °C for 20 minutes.
101. Let the MSA5 plate rest for 30 minutes at room temperature, then centrifuge at 280 xg.
102. Remove the Seal Heat Foil.
103. Remove the BeadChip carefully from the package, do not touch inlets or beadstrips.
104. Preparation of the Beadchip Hyb Chamber by adding 400 µl PB2 buffer for each reservoir in the BeadChip Hyb Chamber.

105. Insert the BeadChip in Hyb Chamber insert, so the barcode is aligned with the barcode symbol on Hyb Chamber insert.
106. Add by pipette 26 µl of each sample to each inlet on the Beadchip. Each beadstrip should be completely covered by sample material.
107. Insert Hyb Chamber Insert loaded with the Beadchip in the BeadChip Hyb Chamber.
108. Carefully attached the BeadChip Hyb Chamber lid, while Hyb Chamber Insert loaded with the Beadchip remains at the accurate position.
109. Incubate at 48 °C for at least 16 to maximum of 24 hours.
110. Remove the BeadChip Hyb Chamber from the incubator and let it rest for 25 minutes at room temperature.
111. Remove the Beadchip from the BeadChip Hyb Chamber and the Hyb Chamber Insert.
112. Remove the seal of the BeadChip without touching the beadstrips.
113. Place the BeadChip in a Coplin jar filled with 60 ml of PB1.
114. Steeping the BeadChip in the Coplin jar for one minute.
115. Place the black frame in the Multi-Sample BeadChip Alignment Fixture containing 150 ml PB1.
116. Place the BeadChip in the black frame aligned by the barcode and the barcode symbol, any seal left on the BeadChip is carefully removed by the tip of a pipette.
117. Place the see-through spacer on top of the BeadChip in the Multi-Sample BeadChip Alignment Fixture.
118. Place a glass-plate over the see-through spacer so that the BeadChip is visible with the barcode up.
119. Place the Alignment Bar in the Multi-sample BeadChip Alignment Fixture so that the Alignment Bar fits the four containers.
120. Attach two metal clips so that the samples are fixed in the BeadChip according to the Alignment Bar.
121. Remove the Alignment Bar and cut the fixed BeadChip ends according to the see-through spacer.
122. Insert the fixed BeadChip in a Chamber Racket in a water circulator heated to 44 °C ± 0,5 °C to prepare for Single base extension.

Single base extension (each reagent added to the plate, through the carving in the glass, in the order described below)

1	150 µl	RA1	30 seconds	X 5
2	450 µl	XC1	10 minutes	
3	450 µl	XC2	10 minutes	
4	200 µl	TEM	15 minutes	
5	450 µl	95 % formamid/1 mM EDTA	1 minute	
6	450 µl	95 % formamid/1 mM EDTA	5 minute	
7	Lower the temperature for the water circulator to 33,5 °C so that the Chamber Racket temperature reaches the temperature noted on the STM tube (32 °C)			
8	450 µl	XC3 (added while the temperature drops)	1 minute	
9	Wait for the temperature of the Chamber racket to reach 32 °C			

123. Turn on Illumina® iScan, for the lasers to stabilize.

124. Staining of the fixed BeadChip:

Each reagent added to fixed Beadchip, through the carving in the glass, in the order described below.

1	250 µl	STM	10 minutes	
2	450 µl	XC3	1 minute	X2
3	Wait for 5 minutes			
4	250 µl	STM	10 minutes	
5	450 µl	XC3	1 minute	X2
6	Wait for 5 minutes			
7	250 µl	STM	10 minutes	
8	450 µl	XC3	1 minute	X2
9	Wait for 5 minutes			
10	250 µl	STM	10 minutes	
11	450 µl	XC3	1 minute	X2
12	Wait for 5 minutes			
13	250 µl	STM	10 minutes	
14	450 µl	XC3	1 minute	X2
15	Wait for 5 minutes			

125. Turn off the water circulator and place the fixed BeadChip horizontally at room temperature.
126. Remove the metal clips.
127. Remove the glass-plate and discharge the see-through spacer.
128. Place the BeadChip in a Coplin jar filled with 60 ml of PB1.
129. Steep the BeadChip in the Coplin jar ten times (make sure to elevate the BeadChip completely from the PB1 solution).
130. Transfer the BeadChip to another Coplin jar filled with 60 ml of XC4.
131. Steep the BeadChip in the Coplin jar ten times (make sure to elevate the BeadChip completely from the XC4 solution).
132. Let the BeadChip rest in the XC4 solution for five minutes.
133. Remove the BeadChip from the Coplin jar and wipe the bottom with an ethanol wet wipe and dry the surface with a clean napkin - carefully so as not to touch the beadstrips.
134. Place the BeadChip in a blue tube rack in a desiccator and let it dry for 50-55 minutes under vacuum. (675 mm Hg ~ 0,9 bar).
135. Slowly release the vacuum and make sure the XC4 coating is dry before further processing.
136. Wipe the bottom of the BeadChip with an ethanol wet wipe and dry the surface with a clean napkin - carefully so as not to touch the beadstrips.
137. Insert the BeadChip to iScan by placing the Beadchip in the blue BeadChip Carrier.
138. Place the BeadChip Carrier in the iScan Reader Tray.
139. If Barcode, Type, and Scan Setting are valid, iScan is ready.
140. Data are exported and controlled in BeadArray Controls Reporter (BACR).
141. These data are controlled in GenomeStudio and exported as idat-files.
