# Supplementary material for: Genome-Wide DNA Methylation Profiling as a Prognostic Marker in Pituitary Adenomas—A Pilot Study
Source: Cancers (Basel). 2024 Jun 13;16(12):2210. doi: 10.3390/cancers16122210 (PMC11201450; doi:10.3390/cancers16122210)
Supplement: Supplementary file 1 [file cancers-16-02210-s001.zip › Protocol for extraction of DNA from formalin fixed paraffin embedded_cg.pdf]

Protocol for extraction of DNA from formalin-fixed paraffin-embedded (FFPE) tissue samples.

1. From each FFPE block, cut one 10 µm slice on a microtome.
2. Transfer each slice to a 1,5 ml Eppendorf tube marked for set sample – if limited tissue, cut two slices and transfer to the same Eppendorf tube.
3. Add 160 µl of Deparaffinization Solution to the Eppendorf tube.
4. Vortex for 10 seconds and centrifuge briefly.
5. Incubate at 56 degrees Celsius (°C) for 3 minutes, then centrifuge briefly and cool to room temperature.
6. For one sample, add a mix of 55 µl RNase-free Water, 25 µl Buffer FTB and 20 µl proteinase K to the tube.
7. Vortex and centrifuge briefly.
8. Incubate at 56 °C for 60 minutes, then set a termomixer to 50 °C.
9. Incubate to 90 °C for 60 minutes.
10. Centrifuge briefly to remove droplets from the lid.
11. Transfer the bottom phase (clear phase) to a new 1,5 ml Eppendorf Safe-Lock microtube. The top phase is discarded.
12. Add a mix of 115 µl RNase-free Water and 35 µl Uracil-DNA glycosylase (UNG) (frozen) to the Eppendorf tube.
13. Vortex and centrifuge briefly.
14. Incubate at 50 °C for 60 minutes.
15. Centrifuge shortly to remove droplets from the lid.
16. Add 250 µl Buffer AL. Vortex thoroughly and centrifuge briefly.
17. Add 250 µl 96% ethanol. Vortex thoroughly and centrifuge briefly.
18. Transfer 700 µl of the content by pipette to a QIAamp UCP MinElute columns placed in a 2 ml collection tube.
19. Centrifuge for one minute.
20. Discard the material in the collection tube and add 500 µl Buffer AW1.
21. Centrifuge for one minute.
22. Discard the material in the collection tube and add 500 µl Buffer AW2.
23. Centrifuge for one minute.
24. Discard the material in the collection tube and add 250 µl 96% ethanol.
25. Centrifuge for one minute.
26. Discard the material in the collection tube and place the QIAamp UCP MinElute columns placed in a new 2 ml collection tube.
27. Centrifuge for one minute.
28. Discard the material in the collection tube.
29. Transfer the QIAamp UCP MinElute columns in a 1,5 ml Eppendorf Safe-Lock microtube.
30. Add 100 µl Buffer ATE and incubate for 5 minutes at room temperature.
31. Centrifuge for one minute and discard the QIAamp UCP MinElute columns.
32. DNA is stored in the Eppendorf tube at -80 °C.
